# Consensus Prediction of Protein Conformational Disorder from Amino Acidic Sequence

**DOI:** 10.2174/1874091X00802010001

**Published:** 2008-01-18

**Authors:** Suresh Kumar, Oliviero Carugo

**Affiliations:** 1 Department of Biomolecular Structural Chemistry, Max F. Perutz Laboratories, Vienna University, Campus Vienna Biocenter 5, A-1030 Vienna, Austria; 2 Department of General Chemistry, Pavia University, Viale Taramelli 12, I-27100 Pavia, Italy

## Abstract

Predictions of protein conformational disorder are important in structural biology since they can allow the elimination of protein constructs, the three-dimensional structure of which cannot be determined since they are natively unfolded. Here a new procedure is presented that allows one to predict with high accuracy disordered residues on the basis of protein sequences. It makes use of twelve prediction methods and merges their results by using least-squares optimization. A statistical survey of the Protein Data Bank is also reported, in order to know how many residues can be disordered in proteins that were crystallized and the three-dimensional structure of which was determined.

## INTRODUCTION

It was recently shown that several proteins do not assume a well defined and stable three-dimensional (3D) structure but are natively unfolded [1]. This was absolutely surprising since unfolded proteins are known to be less stable and soluble *in vitro* and protein misfolding is known to be associated with several conformational diseases, including Parkinson and Alzheimer [2]. However, a considerable fraction of the proteome is constituted by natively unfolded proteins and this fraction seems to be larger in higher organisms than in simpler prokaryotes.

Several techniques to predict conformational disorder in proteins have been designed [3-5] and the performance of many of them is periodically checked, within the CASP initiatives [6], where several blinded predictions are made on targets, the conformational status of which is known only by the CASP organizers and is unknown by the various prediction teams that participate to CASP. In general, it appears that (i) the reliability of these predictions is rather modest and that (ii) different predictions are made by different predictors. The first point is *per se* not surprising, given the intrinsic difficulty of predicting 3D features on the basis of amino acidic sequences. The second point - the inconsistency between different prediction methods - is also not very surprising. In fact, various predictors do not differ only in their algorithms but also in what they define as "conformational disorder" and thus in what they want to predict. For example, in one of the DISEMBL versions [7], all the residues in loops are considered to be conformationally disordered, while in another of the DISEMBL versions, only the residues that were not visible in the crystallographic electron density maps are considered to be disordered. Alternatively, in IUPRED no a priori definition of disorder is used [8]. Despite their limitations, the techniques for predicting conformational disorder are extremely important. Initially, they were designed principally to study the interesting phenome-non of conformation disorder and for large-scale proteome comparisons. Later, it became clear that they have also a series of practical applications, like for example in structural genomics, where they are becoming routine filters in the pipeline of finding suitable targets to be analyzed [3, 4]. In fact, it is obvious that the 3D structure of natively unfolded proteins cannot be determined and that these disordered targets must not be analyzed experimentally by structural biologists.

In this paper we present a consensus method, based on various prediction methods, the performance of which is significantly better than that of each individual predictor. Such a new technique is easily usable with freely available software and is interesting not only for structural genomics initiatives but also for traditional hypothesis-driven structural biology. We also report a statistical survey of the Protein Data Bank that shows the fraction of disordered residues in proteins the crystal structure of which was determined. It appears that a moderate fraction of conformationally disordered residues can be tolerated. About 22% of these crystal structures have more the 5% of the residues that are disordered, though only about 2% of them have more than 20% of the residues in a conformationally disordered status.

## METHODS

### Data

Information about conformationally disordered proteins was taken from the DISPROT database (http://www. disprot.org/) release 3.3 [9], which lists, in FASTA format, 458 proteins that are known, on the basis of several experimental studies, to be at least partially disordered. Data were downloaded in August 2006. Each residue of these 458 proteins is labeled according to its conformational status: ordered, disordered, unknown. The main advantage of the DISPROT database is that it is curated by experts and it is not based on some automatic procedure. It is thus reasonable to suppose that it contains a very limited number of inaccuracies.

### Individual Predictors

12 individual predictors were used (Table **1** Some of them are different versions of the same basic algorithm. For example, IUPRED has two versions, one specialized in predicting short disordered polypeptide fragments and the other focused on the prediction of long disordered polypeptide fragments. Others have even three versions, like DISEMBL, which can predict if a residue is in a loop, in a "hot" loop (characterized by high crystallographic B factors), or if it was not observed in the electron density maps, as stated on the “REMARK 465” lines of the files of the Protein Data Bank. Given that the present manuscript is not focused on a particular type of disorder but it is focused on the identification of protein constructs that cannot be studied by structural biologists, we did not make any difference between the various versions of the predictors and we used all of them. This is justified by the fact that we do not want to design a new predictor but we want only to make consensus predictions that can be useful in structural biology for high-throughput structural genomics initiatives and, more in general, in any structural biology project. Moreover, the mathematical approach we used (see below) is essentially unaffected by the use of similar or redundant prediction methods given that it is a least-squares optimization, which by definition, weights all contributions as a function of each other.

### Consensus Predictions

Each prediction method (Table **1)** produces binary results: a residue can be predicted to be conformationally ordered or disordered. From a numerical perspective, this can be represented by a value of +1 if it is predicted to be disordered, or by a value of -1, if it is predicted to be ordered. The numerical value of 1 and its sign, positive or negative, are purely arbitrary and different values or opposite signs would not affect the quality of the results.

P⋅X=D

where **P** is a N x 12 matrix the elements p_ij_ of which are either +1, if the i^th^ residues is predicted to be disordered by the j^th^ prediction method, or -1 if it is predicted to be ordered, and where **D** is a vector of N elements d_i_, the values of which can be either +1, if the i^th^ residues is disordered in the reality, or -1, in the opposite case. The value of N is the total number of residues that are annotated to be ordered or disordered in the DISPROT database and is equal to 54012 residues.

Once the optimal values of the elements of **X** have been determined, it is possible to use them to predict if a residue is conformationally ordered or disordered by computing its *p_cons* value

$P\_\mathrm{cons}=\sum_{i=1}^{12}x_{i}\cdot p\_{\mathrm{indi}}_{i}$

where the values of *p_indi_i_* are either +1, if the residue is predicted to be disordered by the i^th^ prediction method, or -1, if it is predicted to be ordered. If *p_cons* is closer to +1 than to -1, which means if it is greater than 0, the residue is predicted to be disordered. On the contrary, it is predicted to be ordered if *p_cons* 0. The optimal values of the coefficients x_i_ are reported in Table **2**.

### Prediction Validation

Given the extremely high number (54012) of amino acid residues contained in the DISPROT database, a complete cross-validation, known also as Jack-knife test, is impossible. We performed thus a 20-fold cross-validation: we built randomly 20 non-overlapping sets of residues, each containing 5% of the data, and the optimization of the **X** vector was performed 20 times by discarding each time one of the small subsets, which was then used to compute the *p_cons* values. Such a separation between the learning sets and the test sets allows one to make unbiased predictions, which can then be compared with the experimentally known conformational statuses of the residues.

A residue correctly predicted to be disordered was counted as a true positive (tp). A residue correctly predicted to be ordered was counted as a true negative (tn). A disordered residue predicted to be ordered was counted as a false negative (fn). An ordered residue predicted to be disordered was counted as a false positive (fp). Given these four quantities, the prediction reliability was estimated with a series of figures of merit: the sensitivity, the specificity, the accuracy, and the probability excess, defined as

$\mathrm{sensitivity}=\frac{\mathrm{tp}}{\mathrm{tp}+\mathrm{fn}}$

$\mathrm{specificity}=\frac{\mathrm{tp}}{\mathrm{tp}+\mathrm{fp}}$

$\mathrm{specificity}=\frac{\mathrm{tp}+\mathrm{tn}}{\mathrm{tp}+\mathrm{tn}+\mathrm{fp}+\mathrm{fn}}$

probability_excess=sensitivity+specificity−1

The values of these figures of merit can range from 0 to +1 and larger values, closer to +1, are associated with better predictions. It must be observed that some of these figures of merit, typically the accuracy, can be seriously biased if the data are unbalanced. This is exactly what happens here, since the number of ordered residues (2649) is very different from the number of disordered residues (51363) in the database DISPROT. The values of accuracy are thus provided in the present paper only because this figure of merit is used very commonly in computational biology. A much more robust indicator of prediction quality is the probability excess.

## RESULTS AND DISCUSSION

Besides their basic biological importance, predictions of protein conformational disorder are important in structural biology, where "impossible" targets must be identified before inserting them in the experimental pipe-line that goes from cloning to structural determination. This is particularly important not only in structural genomics initiatives, the success rate of which is still rather modest, but also in traditional hypothesis-driven applications, especially when the protein construct must be designed by the scientists, like for example in multi-domain protein and viral poly-proteins [10].

Predictions of conformational disorder are thus one of the bioinformatics filters that must be used before moving towards experimental analyses. Other filters are focused on the quaternary structural requirements of a protein chain [11], on protein solubility and stability [12, 13], and some web-based servers were created to assist the users in this task [14, 15].

However, before doing predictions of conformational disorder it is necessary to know what level of disorder can be tolerated by well folded proteins. In fact, while it is clear that the 3D structure of a completely disordered protein cannot be determined, it is also clear that many (or, maybe, most) proteins are partially disordered.

For example, many loops at the protein surface are very flexible and tend to adopt more than a single shape. For this reason, we scanned the Protein Data Bank (PDB) [16, 17] looking for regions conformationally disordered.

This information was extracted from the records labeled with "REMARK 465", where the depositors of the crystal structures declare, if necessary, which residues were not observed in the electron density maps. This analysis was limited to the crystal structures, which are nevertheless the large majority of the entries of the PDB, and it was assumed that the location of completely unfolded segments cannot be detected in the electron density maps.

**Fig. (1):**
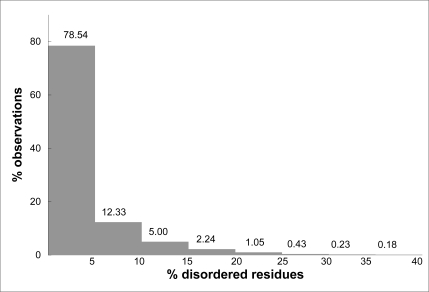
Distribution of protein crystal structures as a function of the percentage of disordered residues they contain. The data were taken from the Protein Data Bank; a residues was considered to be disordered if not observed in the crystallographic electron density maps; the total number of residues was taken from the SEQRES record of the PDB files.

Fig.**1**) shows the distribution of the PDB entries according to their fraction of residues not observed which are likely to be conformationally disordered. It appears that a considerable number of structures have conformational disorder. In 22% of them, more than 5% of the residues are disordered. However, only about 2% of the crystal structures contain more than 20% of the residues that lack a well defined structure. The most extreme case is the entry 1VCR, the light-harvesting complex from *Pisum sativum* thylacoid membrane, where 56% of the residues were not observed, though this crystal structure was determined and refined at very low resolution (9.5 Å) [18].

**Fig. (2):**
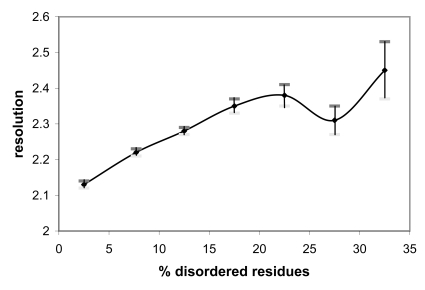
Dependence between the crystallographic resolution and the percentage of disordered residues observed in the crystal structures deposited in the Protein Data Bank. Vertical bars indicate the standard deviation of the mean.

Fig. **2**) shows the relationships between the crystallographic resolution and the percentage of disordered residues. It can be seen that resolution tends to decrease if the amount of disorder increases, though the effect of disorder on resolution is not spectacular. In fact the average resolution decreases only from 2.13 to 2.45 Å if the disorder fraction increases from 2.5 to 32.5%.

This clearly shows that protein 3D structures are often partially disordered and that a moderate fraction of conformationally disordered residues can be tolerated. Keeping this in mind, one can now try to predict if a protein has a reasonable probability to be suitable for a structural biology analysis. We designed a prediction method that is based on several individual prediction algorithms. The only necessary input is the amino acidic sequence of the protein and all the predictors are freely available. Each prediction algorithm must be used separately (Table **1**) and its results must be inserted into equation (2), together with the coefficients x_i_ reported in Table **2**. If the value of *p_cons* is positive, the residue is predicted to be disordered and if it smaller than zero, the residue is predicted to be ordered. This can easily be done for each residue and, as a consequence, it is possible to reach a global picture of the conformational status of the protein.

This new prediction method, which is essentially a weighted consensus approach, performs quite well, better than any individual prediction algorithm. Table **3** shows the values of several figures of merit, obtained with a 20-fold cross validation procedure. It can be seen that predictions are very accurate, with all the figures of merit larger than 80%. This is impossible by using individual predictors, though all of them have very high specificity. The probability excess, which is the best figure of merit because little influenced by the fact that the data are unbalanced, is equal to 80.1%, a value much larger than any other predictor.

It must be observed that the prediction reliability described above is based on the particular set of proteins available at the DISPROT database. Therefore, it would not be surprising to obtain other estimations of reliability by using different data.

As a consequence, the reliability indicators shown in Table **3** cannot be used to rank various prediction methods according to their performances. It is however clear that the consensus approach presented in this manuscript is likely to be superior to all the individual methods on which it is based and it is also reasonable to suppose that an increase of experimental knowledge, which is likely to occur in the future, will allow more accurate predictions.

## Figures and Tables

**Table 1:** Individual Prediction Methods Used in the Present Paper

Method	URL	reference
DISEMBL_hot_loops	http://dis.embl.de/	[7]
DISEMBL_loops	http://dis.embl.de/	[7]
DISEMBL_remark465	http://dis.embl.de/	[7]
DISOPRED	http://bioinf.cs.ucl.ac.uk/disopred/	[19]
DRIPRED	http://www.sbc.su.se/~maccallr/disorder/	[20]
FOLDINDEX	http://bip.weizmann.ac.il/fldbin/findex	[21]
GLOBPLOT_B	http://globplot.embl.de/	[22]
GLOBPLOT_r	http://globplot.embl.de/	[22]
IUPRED_L	http://iupred.enzim.hu/	[8]
IUPRED_S	http://iupred.enzim.hu/	[8]
PRELINK	http://genomics.eu.org/spip/PreLink	[23]
RONN	http://www.strubi.ox.ac.uk/RONN	[24]

**Table 2:** Optimal Values of the Coefficients xi to be Used to Compute the *p_cones* Values (Equation 2)

Method	x
DISEMBL_hot_loops	-0.101
DISEMBL_loops	0.377
DISEMBL_remark465	-0.172
DISOPRED	0.048
DRIPRED	0.096
FOLDINDEX	0.262
GLOBPLOT_B	-0.199
GLOBPLOR_r	0.162
IUPRED_L	0.041
IUPRED_S	-0.126
PRELINK	0.078
RONN	0.141

**Table 3:** Performance of the New Prediction Methods Described in the Present Paper Compared to the Individual Prediction Methods of Table 1

Method	sensitivity	specificity	accuracy	probability excess
Consensus	0.833	0.968	0.814	0.801
DISEMBL_hot_loops	0.481	0.974	0.494	0.455
DISEMBL_loops	0.761	0.966	0.747	0.727
DISEMBL_remar465	0.409	0.977	0.428	0.385
DISOPRED	0.568	0.994	0.586	0.562
DRIPRED	0.640	0.975	0.642	0.615
FOLDINDEX	0.688	0.981	0.691	0.669
GLOBPLOT_B	0.421	0.990	0.445	0.410
GLOBPLOR_r	0.589	0.979	0.597	0.568
IUPRED_L	0.609	0.993	0.624	0.602
IUPRED_S	0.529	0.996	0.550	0.524
PRELINK	0.512	0.970	0.521	0.483
RONN	0.634	0.985	0.642	0.618
